# An Optimized Method for Evaluating the Preparation of High-Quality Fuel from Various Types of Biomass through Torrefaction

**DOI:** 10.3390/molecules29081889

**Published:** 2024-04-21

**Authors:** Shuai Guo, Xiaoyan Deng, Deng Zhao, Shujun Zhu, Hongwei Qu, Xingcan Li, Yan Zhao

**Affiliations:** 1 School of Energy and Power Engineering, Northeast Electric Power University, Jilin 132012, China; guoshuaidq@126.com (S.G.); d477757570@163.com (X.D.); xingcanli@neepu.edu.cn (X.L.); 2 Shanxi Key Laboratory of Coal Flexible Combustion and Thermal Conversion, Datong 037000, China; zhushujun@iet.cn; 3 College of Vehicles and Energy, Yanshan University, Qinhuangdao 066000, China; zhaodeng0810@sina.com; 4 Shenyang Academy of Environmental Sciences, Shenyang 110167, China

**Keywords:** biomass, torrefaction, displacement level, solid biofuel

## Abstract

The pretreatment for torrefaction impacts the performance of biomass fuels and operational costs. Given their diversity, it is crucial to determine the optimal torrefaction conditions for different types of biomass. In this study, three typical solid biofuels, corn stover (CS), agaric fungus bran (AFB), and spent coffee grounds (SCGs), were prepared using fluidized bed torrefaction. The thermal stability of different fuels was extensively discussed and a novel comprehensive fuel index, “displacement level”, was analyzed. The functional groups, pore structures, and microstructural differences between the three raw materials and the optimally torrefied biochar were thoroughly characterized. Finally, the biomass fuel consumption for household heating and water supply was calculated. The results showed that the optimal torrefaction temperatures for CS, AFB, and SCGs were 240, 280, and 280 °C, respectively, with comprehensive quality rankings of the optimal torrefied biochar of AFB (260) > SCG (252) > CS (248). Additionally, the economic costs of the optimally torrefied biochar were reduced by 7.03–19.32%. The results indicated that the displacement level is an index universally applicable to the preparation of solid fuels through biomass torrefaction. AFB is the most suitable solid fuel to be upgraded through torrefaction and has the potential to replace coal.

## 1. Introduction

Fossil fuels dominate the global energy sector, accounting for as much as 80%. However, owing to their nonrenewable nature and severe environmental impacts, there is an urgent need to seek new energy alternatives to increase the supply of renewable energy and reduce greenhouse gas emissions [1,2]. Biomass is the only carbon-neutral renewable energy source and has advantages such as large reserves, environmental friendliness, and near-zero CO_2_ emissions [3]. However, biomass feedstocks have some limitations, such as high moisture content, high oxygen content, poor grindability, low heating value, and hygroscopicity [4]. To overcome these challenges and achieve the efficient energy conversion and resource utilization of biomass, an effective pretreatment method is required. Torrefaction, as a mild thermal decomposition process, improves the uniformity and grindability of biomass feedstocks and increases their energy density by low-temperature heating (200–300 °C) under an atmospheric inert atmosphere while eliminating volatile acid content [5,6]. Biomass treated via torrefaction can produce carbon-rich solid fuels, significantly enhancing the quality of solid biomass fuels [7]. Overall, biomass has enormous potential as an alternative to fossil fuels and torrefaction is a promising treatment method that can significantly improve the availability and energy value of biomass feedstocks. This is of great significance for reducing our dependence on nonrenewable energy sources, lowering greenhouse gas emissions, and protecting the environment.

Many researchers have studied the effects of torrefaction pretreatment on the physicochemical properties of biochar. Simonic et al. found in their study on wood torrefaction that torrefaction temperature is the most crucial among many influencing factors [8]. Li et al. [9] and Kanwal et al. [10] reached the same conclusion in their studies on the torrefaction of tomato residues and sugarcane bagasse. Chen et al. [11] discovered in their study on rice husk torrefaction that the optimal reaction temperature range for rice husk is 230–260 °C. Ivanovski et al. [12] found that with increasing torrefaction temperature, the oxygen content of the products decreased continuously, the heating value increased, and the grindability of the biochar was significantly enhanced compared to that of the untreated sample. Yang et al. [13] found in their wheat straw torrefaction study that the higher the torrefaction temperature, the greater the heating value of the product. When the temperature reached 300 °C, the heating value increased from the initial 12.88 MJ/kg to 16.08 MJ/kg, and the reaction activation energy decreased from the initial 174.89 kJ/mol to 129.3 kJ/mol, significantly enhancing the reactivity of the char. In their study on sludge torrefaction, Ahmad et al. [14] found that with increasing torrefaction temperature, the fixed carbon content of sludge char increased by 47.79% and the moisture content decreased by 61.35% at 300 °C. In their study on spent coffee grounds (SCGs), Nepal et al. [15] determined 290 °C to be the optimal torrefaction temperature, resulting in a 36.04% increase in heating value compared to untreated raw material. Mukhtar et al. [16] investigated energy densification through the torrefaction of corncobs and rice husks and found that the optimal energy densification was achieved at a temperature of 250 °C and a duration of 30 min. The energy yield of torrefied corncobs was 115.32%, which was 19.62% higher than that of rice husks, increasing its energy density from 1.20 to 1.48. Garba et al. [17] studied the effects of torrefaction on the characteristics of peanut shells, rice husks, and corncobs and found that the intensity of the functional group peaks increased with increasing torrefaction temperature. During torrefaction, rice husks and corncobs exhibited higher weight loss, whereas peanut shells showed the strongest thermal stability, followed by corncobs and rice husks. In recent years, many optimization methods have been applied to biomass torrefaction pretreatment techniques to better understand the effects of torrefaction conditions on biomass fuel characteristics. Aniza et al. [18] conducted microwave torrefaction experiments on waste mushroom substrates using orthogonal experimental methods and investigated the effects of the sample size, catalyst, and power on the fuel yield. They observed the highest total fuel yield at 335 μm particle size, 300 mg catalyst, and 900 W power. Brotto et al. [19] optimized the torrefaction process of wood chips through a two-factor experiment, with the results indicating that the optimal product properties were achieved at a temperature of 290 °C and a reaction time of 30 min. Singh et al. [20] analyzed the influence of the torrefaction temperature and reaction time on the higher heating value (HHV) of eucalyptus using response surface methodology (RSM) and a central composite design. The optimal torrefaction charcoal was obtained at a temperature of 280 °C and a time of 60 min, resulting in an increase in calorific value and energy density by 37.1% and 12.9%, respectively, compared to the raw sample. In addition, the fuel ratio and combustibility index increased by 43.3% and 76.2%, respectively. Guo et al. [21] optimized the calorific value and yield of corn stover (CS) char using RSM and the Box–Behnken design. They found that at a reaction temperature of 242.26 °C, residence time of 60 min, and heating rate of 6.28 °C/min, the retention rate increased by 13.75%. Guo et al. [22] conducted a multi-objective optimization on the torrefaction of agaric fungus bran (AFB) and waste plastic, finding that the optimal conditions for simultaneously optimizing the quality yield–heat and value–ash content were 230.68 °C, 30 min, and a mixing ratio of 20%, while for simultaneously optimizing the energy yield-ash content, the optimal conditions were 220 °C, 30 min, and a mixing ratio of 20%. These results indicate a reduction in the ash content of 15.71% and 14.88%, respectively, while maintaining a high yield and HHV. Viegas et al. [23] optimized the torrefaction of mixed biomass of microalgae and lignocellulosic biomass using RSM, finding that at 250 °C, 60 min, and 50% lignocellulosic biomass, the highest yield and HHV of the mixed biochar were achieved, at 76.5% and 17.4 MJ/kg, respectively. In another study [24], the torrefaction process of olive waste was optimized using a numerical modeling method, with the optimal operating conditions being 275 °C and 30 min, resulting in an 18.18% increase in HHV. Based on the above research results, it can be concluded that the optimal torrefaction temperature for lignocellulosic biomass falls between 260–300 °C.

While previous research on biomass torrefaction pretreatment has been relatively extensive, discussions have predominantly focused on individual samples, whether single biomasses or blends were used, and whether optimized methods were employed. However, such discussions often fail to provide a comprehensive comparison of the characteristics of various types of biomass fuels. Consequently, performing horizontal comparisons across different types of biomass fuels is challenging. Therefore, a method to evaluate and compare the overall characteristics of multiple biomass fuels using a certain method is vital to enhance the efficiency of torrefaction pretreatment and increase the accuracy of biomass resource energy conversion.

This study employed the “displacement level” indicator [25] to optimize the fuel characteristics of different types of biomass torrefaction products. This is a novel comprehensive evaluation indicator for fuels that allows a comprehensive assessment of fuel properties and horizontal comparisons. We conducted fluidized bed torrefaction experiments on three different biomass feedstocks (CS, AFB, and SCGs) in a batch reactor and analyzed the effect of torrefaction temperature on the properties of the different types of biomass fuels. We determined the optimal operating temperatures, conducted detailed characterization, and established the relationship between the microstructure and optimal conditions. To the best of our knowledge, this is the first study to comprehensively analyze the optimization of torrefaction biochar using the “displacement level” indicator.

## 2. Results and Discussion

### 2.1. Physicochemical Properties of Biochar Samples

Table 1 presents the yield, calorific value, and energy density of the CS, AFB, and SCGs at different torrefaction temperatures. As the torrefaction temperature increased, the mass yield of all three types of biomass decreased, primarily because of the thermal degradation and devolatilization of hemicellulose. Hemicellulose exhibits more active thermal degradation at high temperatures compared to cellulose and lignin, with a sharp decrease in mass yield observed at 280 °C, retaining only half of its initial mass [26,27]. However, it is worth noting that AFB had the highest mass yield, followed by SCGs and CS, which was attributed to the higher content of easily degradable components in SCGs and CS. Additionally, the energy yield exhibited a behavior similar to that of the mass yield, with the rate of decrease in the energy yield being smaller than that of the mass yield. This indicates that the energy yield is influenced more by the mass yield, further predicting the positive role of torrefaction in enhancing the sustainability of HHV and combustion [28]. Although the biomass yield decreased with increasing torrefaction temperature, its HHV and energy density exhibited increasing trends, especially at higher temperatures. The HHV and energy density of the CS, AFB, and SCGs increased by 17.1%, 23.9%, and 23.8%, respectively, and by 13.2%, 13.8%, and 14.8%, respectively. This phenomenon may be attributed to the relationship between HHV and biomass degradation, with lignin contributing the most to HHV. Alternatively, an increase in temperature promotes decarboxylation and dehydration, thereby increasing the carbon content of the biochar and consequently increasing its HHV and energy density [29].

**Figure 1:**
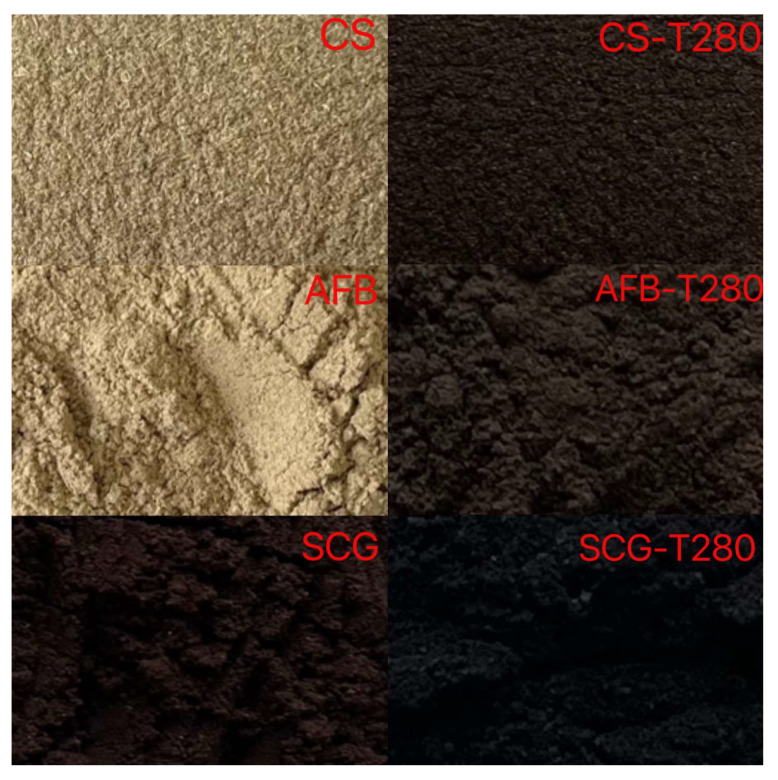
Samples of the three biomasses before and after torrefaction.

Additionally, the biomass appeared darker and more brittle after torrefaction than in its original state, as shown in Figure 1. This was attributed to changes in the fiber properties during torrefaction, leading to a decrease in fiber toughness and an increase in brittleness. This indicates that less grinding energy was required for post-torrefaction, resulting in improved grindability [30].

**Figure 2:**
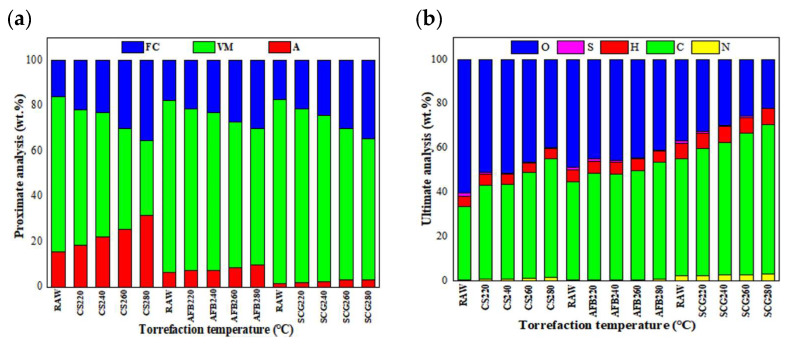
Proximate and ultimate analyses of the three biomasses before and after torrefaction. Note: (**a**) FC: Fixed Carbon, VM: Volatile matter, A: Ash, (**b**) O: Oxygen, S: Sulfur, H: Hydrogen, C: Charcoal, N: Nitrogen.

Comparison of proximate and ultimate analyses as shown in Figure 2a, with an increase in the torrefaction temperature, the volatile matter content significantly decreased (20.7–52%), whereas the fixed carbon content increased (68.5–121%). This is attributed to the decomposition of biomass components into volatile or gaseous products such as hydrogen, water, carbon dioxide, carbon monoxide, furfural, methanol, formaldehyde, acetic acid, and formic acid at higher torrefaction temperatures, leading to a relative increase in lignin content during pyrolysis [31]. Sakulkit et al. [32] found that samples with higher fixed carbon contents had higher energy densities and calorific values, which is consistent with our research findings. Additionally, the ash content of all samples increased with increasing torrefaction temperature. Compared with CS, the increase in the ash content of AFB and SCGs was smaller as the temperature increased. Ash is an incombustible mixture of metal oxides that not only reduces the operational efficiency of boiler equipment but also leads to slagging and corrosion, thereby decreasing the lifespan of boilers. Therefore, selecting samples with lower ash content as solid fuels is worth considering [33,34]. From Figure 2b, it can be observed that the main components of CS, AFB, and SCGs were carbon and oxygen, constituting over 80% of the basic elemental composition of biomass, whereas the sulfur (1.05–1.75%), nitrogen (0.42–2.38%), and hydrogen (4.53–7.07%) contents were relatively low. With an increase in torrefaction temperature, the chemical composition of the biomass samples changed, with the decrease in O and H content mainly attributed to the release of gases such as H_2_O, CO_2_, and CO, resulting in a relatively lower O content corresponding to a higher HHV. Meanwhile, the carbon content increased with an increase in the torrefaction temperature, with SCGs exhibiting a significantly higher carbon content than the other two biomasses. The carbon content of SCG-T280 reached 68%, which was similar to that of bituminous coal and corresponded to the highest HHV. Additionally, the nitrogen content of AFB was relatively low, indirectly indicating that charcoal from AFB may reduce NO_x_ emissions and minimize coke and smoke generation in future thermal conversions [35].

**Figure 3:**
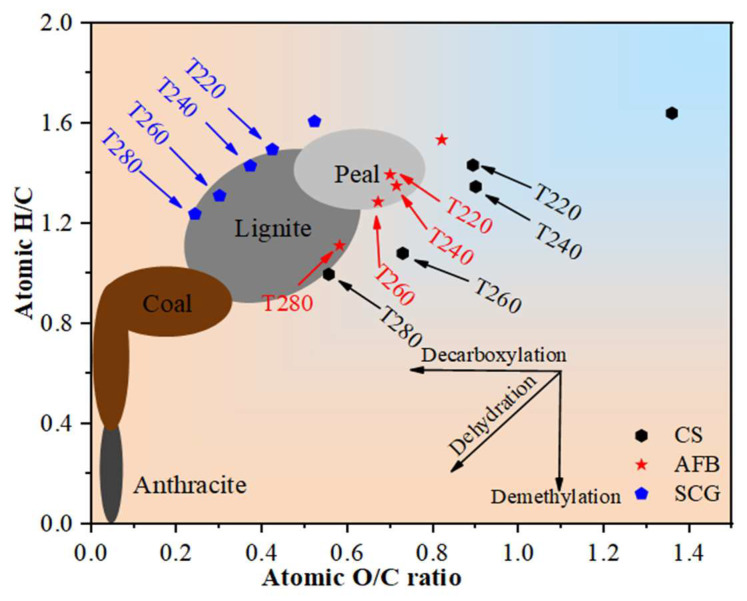
The van Krevelen diagrams of the three biomasses before and after torrefaction, showing the atomic ratios of H/C and O/C.

The van Krevelen diagram is an effective method for elucidating the characteristics of solid fuels, as illustrated by the H/C and O/C ratios of different fuels in Figure 3. Compared to the raw samples, all the torrefied chars exhibited a decrease in the H/C and O/C ratios, which was attributed to dehydration, decarboxylation, and decarboxylation reactions during torrefaction. Lower H/C and O/C ratios of carbonization and better stability indicate a higher degree [12]. Notably, the most significant decrease in the H/C and O/C atomic ratios occurred in CS-T240, with reductions of 18% and 34%, respectively, possibly owing to the high cellulose content in CS [36] Additionally, the changes in the H/C and O/C atomic ratios for AFB were relatively gradual at low torrefaction temperatures but exhibited a significant decrease at higher temperatures, decreasing by 27% and 29%, The relationship between the H/C and O/C atomic ratios of both raw and torrefied coffee grounds showed a linear trend, with reductions of 22% and 53%, respectively, consistent with the findings of Zhang et al. [37]. This study suggests that when biomass is torrefied at temperatures above 240 °C, its properties are similar to those of peat and lignite.

### 2.2. Normalized Assessment of Various Raw Material Torrefied Biochar

#### 2.2.1. Analysis of Pyrolysis Behavior Characteristics

**Figure 4:**
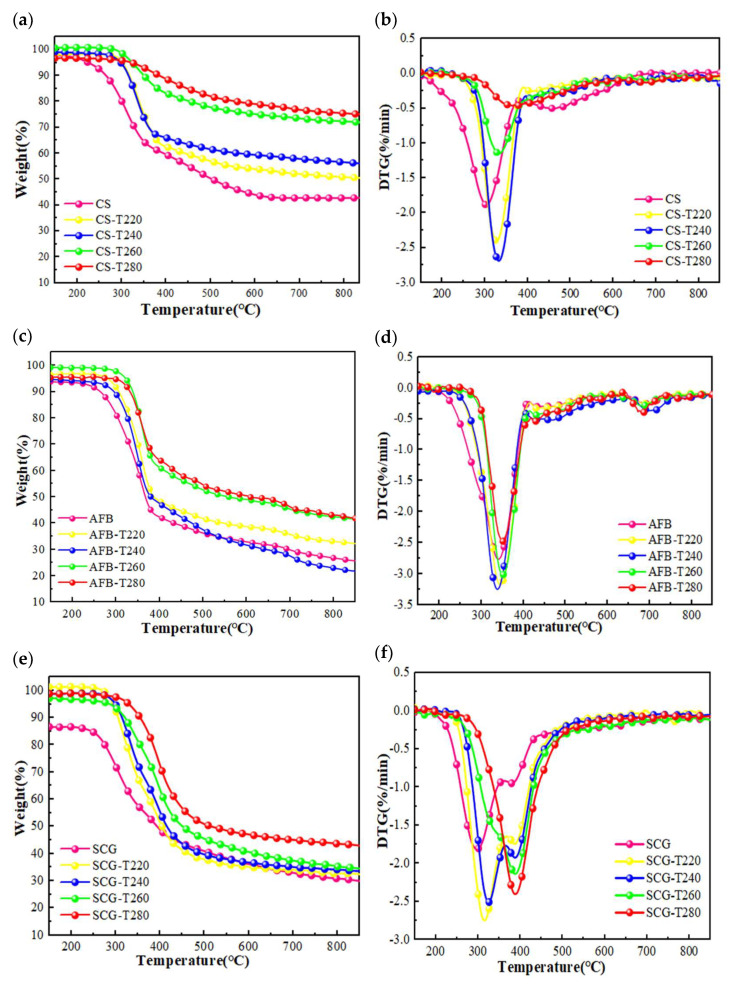
(**a**–**f**) TG/DTG of the three biomasses before and after torrefaction.

TGA was conducted to study the pyrolysis behavior of the CS, AFB, and SCG samples. The corresponding TG and DTG curves are shown in Figure 4. For all the biomass types, the mass loss of the samples gradually decreased as the degree of torrefaction increased. This was attributed to the release of some organic components in the form of volatiles during torrefaction, which originated mainly from hemicellulose and partially from cellulose [38]. While the pyrolysis behavior of biomass at different torrefaction temperatures varies slightly, pyrolysis can generally be divided into three stages: initial decomposition, main decomposition, and final decomposition. The first stage occurred between room temperature and 220 °C, where there was minimal change in sample weight due to the evaporation of moisture and the volatile release of AAEMs. The second stage occurs between 260 and 520 °C, where there is a significant reduction in weight loss, primarily due to the intense thermal decomposition of cellulose. The third stage occurs above 520 °C and involves the slow thermal decomposition of residual lignin, resulting in the production of fixed carbon [39]. For the CS feedstock, the residual mass fraction was 42.6%, but with an increase in the torrefaction temperature, it gradually increased until it reached 73%, which was attributed to the formation of more biochar through cross-linking reactions during torrefaction [40]. From Figure 4b, it is evident that CS exhibits a lower weight loss than torrefied char. Specifically, at around 300 °C, the maximum weight loss rate of raw CS is 1.9%/min, while at approximately 330 °C, CS-T240 has a maximum weight loss rate of 2.7%/min. Figure 4c illustrates the weight loss curve of the Auricularia auricula sample, which is similar to the CS sample. At approximately 340 °C, the maximum weight loss rate of AFB was 2.7% per minute (Figure 4d). In contrast, the weight loss peak of AFB char residues shifts to the left to around 335 °C, with a maximum weight loss rate of 3.22% per minute. Except for SCG-T280, all the DTG curves of SCGs exhibited noticeable shoulder peaks. The prominent weight loss peaks were mainly due to the differences in the cellulose, hemicellulose, and lignin contents.

#### 2.2.2. Assessment of Displacement Level

**Figure 5:**
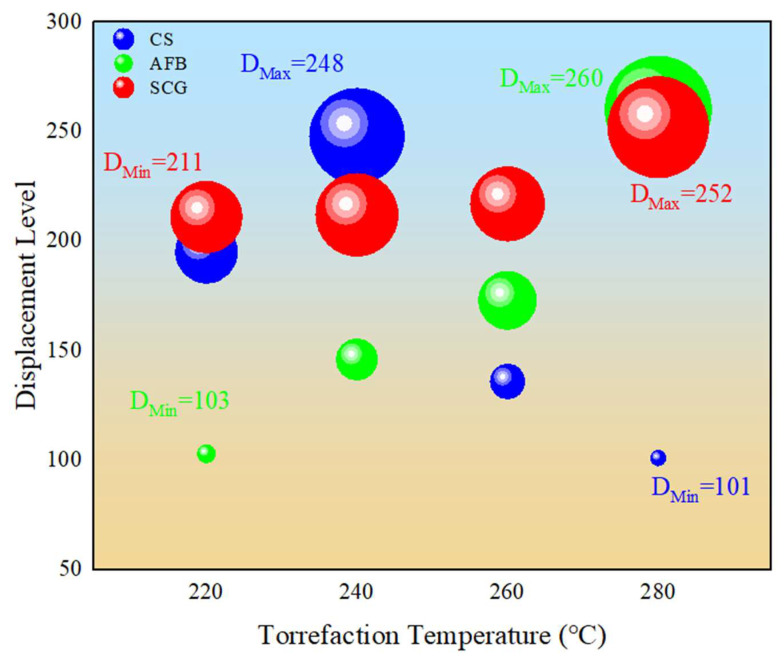
Displacement level of various biochars.

To comprehensively evaluate the fuel properties and achieve cross-sample comparison, we calculated the “Displacement Level” values for each sample based on the DTG results, as shown in Figure 5. The displacement level of the CS exhibited a trend of initially increasing and then decreasing with torrefaction temperature. It peaked at 248 at 240 °C and decreased to 101 at higher torrefaction temperatures. We speculate that this is directly related to the fuel quality. Compared to the mass yield of CS at 240 °C, the level at 280 °C decreased by 28.05%, while the HHV increased by 8.82%. At 280 °C, the ash content significantly increased to 43.56%. The comparison revealed that the positive benefits of increasing the torrefaction temperature for CS were significantly lower than its negative impacts, resulting in a decrease in the displacement level. Therefore, 240 °C is the optimal torrefaction temperature for CS, consistent with the conclusion drawn by Guo et al. [21] through the RSM optimization of the CS torrefaction process. The displacement level of AFB increases monotonically with the torrefaction temperature, rising from an initial value of 103 to 260, indicating that higher torrefaction intensity can provide better fuel quality. The results indicate that at 280 °C, the AFB achieved its maximum energy density of 1.24 when the mass retention rate was 63.62%. For SCGs, the overall trend was not as pronounced. The displacement level peaked at 252 at 280 °C. It is noteworthy that SCGs already reached a displacement level of 211 at low torrefaction temperatures (220 °C). At higher reaction temperatures, the change in the displacement level was not significant, with a maximum upgrade rate of only 19%. We speculate that this may be due to the inherently good fuel characteristics of SCGs. Comparatively, its original HHV was 23.03 MJ/kg, which increased to 28.51 MJ/kg at 280 °C. This indicates that more heat can be released during combustion, which results in a high energy efficiency [41]. SCGs exhibit relatively ideal H/C and O/C ratios, contributing to better combustion efficiency and stability [22]. Overall, the ranking of the optimal torrefaction charcoal fuel characteristics for the three selected biomasses in this study is as follows: AFB-T280 (260) > SCG-T280 (252) > CS-T240 (248). Torrefaction pretreatment had the most significant enhancement effect on AFB, increasing it by 152%, whereas the enhancement of SCGs was the lowest, increasing by only 19%.

Furthermore, we compared the EY, HHV, H/C, and O/C of the biochar produced under optimal torrefaction conditions with those of other biochars (cotton stalks, olive waste, rice husk, and oil palm frond) (see Table 2). It can be observed that under low-temperature torrefaction conditions with relatively low energy consumption, these three types of biomass exhibited comparable or even higher fuel quality compared to other biochars. The research findings suggest that biochar produced through torrefaction has significant potential as a renewable energy source.

### 2.3. Microstructural Analysis of Biomass Raw Material and Optimal Torrefied Biochar

**Figure 6:**
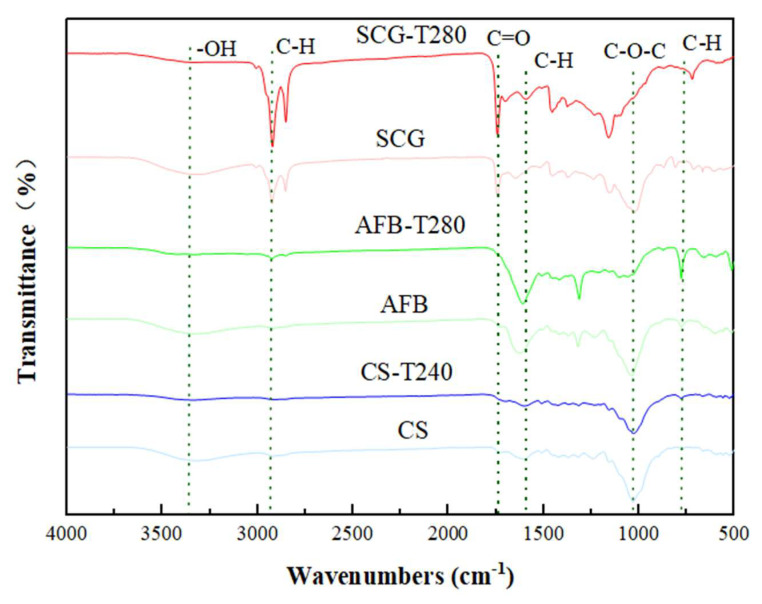
Compositions of raw and optimal samples.

The Fourier-transform infrared spectroscopy (FTIR) is extensively employed to characterize structural alterations in biomass. This is because it can discern the existence of fundamental molecular vibrations that act as distinctive chemical constituents or compound categories. Additionally, it can detect the presence of functional groups like alkenes, esters, aromatics, ketones, and alcohols in biomass. Consequently, it serves as a potent tool for characterizing the micro-mechanisms of pyrolysis [45,46]. Therefore, the functional groups of the three biomass feedstocks and their respective optimally torrefied biochars were determined via FTIR (Figure 6).

The peaks between 3000 and 2800 cm^−1^ are associated with the stretching vibrations of −CH_3_ and −CH_2_ in fatty hydrocarbons or cycloalkanes present in cellulose and hemicellulose [47,48]. The peaks between 1700 and 1600 cm^−1^ are attributed to the stretching vibrations of the carbonyl (C=O) groups in hemicellulose or esters and the stretching vibrations of the aromatic rings (C=C) present in lignin [45]. The absorption peaks between 1600 and 1100 cm^−1^ represent the stretching vibrations of bonds such as C−O−C, C−H, and C−OH. Peaks between 1000 and 650 cm^−1^ represent the out-of-plane bending vibrations of the C−H groups in the aromatic rings [49]. Although the spectra of the three biomass samples and their optimally torrefied chars were similar, there were some differences in the intensity. For example, under optimal torrefaction conditions, certain functional groups in CS may weaken or even disappear. The weakening of the C=O bond absorption peak at 1607 cm^−1^ was attributed to the decomposition of hemicellulose and lignin, resulting in a reduction in the peaks of hydrophilic oxygen-containing functional groups. This reduction led to a decrease in the polar oxygen-containing groups on the surface of the torrefied char, thereby reducing its hydrophilicity [50]. Additionally, the absorption peak at 1025 cm^−1^ gradually weakened, possibly because of the breaking of C−O−C, C−H, and C−OH bonds in cellulose and hemicellulose under torrefaction conditions, leading to the formation of ketones and furan compounds [51]. Compared to AFB, the C=O absorption peak at 1610 cm^−1^ of AFB−T280 was stronger than that of CS−T240 but also weakened. The C−O−C absorption peak at 1031 cm^−1^ was almost absent, indicating that at higher temperatures, the low−cellulose and hemicellulose components were greatly affected. The reduction in hemicellulose and lignin enrichment enhanced the uniformity of the carbonized samples [52]. Under optimal roasting conditions, the SCGs exhibited enhanced absorption peaks of the C−H bond at 2924 and 2850 cm^−1^. Similar peaks were observed in the FTIR spectra of the tea, coffee, and caffeine-containing beverages in the corresponding regions. This is attributed to the asymmetric stretching of the methyl (−CH_3_) C−H bonds in the caffeine molecules [53]. The absorption peaks of the C=O bond at 1740 and 1644 cm^−1^ decreased after roasting owing to the decarboxylation reactions. Additionally, the absorption peaks of the C−O−H bond at 1155 and 1025 cm^−1^ weakened, which was attributed to the thermal decomposition of sugar aldehyde ester groups in the fiber structure during torrefaction [54]. Compared to CS, the disappearance of the -OH absorption peak at 3328 cm^−1^ was more pronounced in AFB−T280 and SCG−T280, indicating intense dehydration reactions of cellulose and hemicellulose in AFB and SCGs during torrefaction, which, in turn, significantly improved the hydrophobicity of AFB and SCG biochar [55], consistent with the aforementioned ultimate analysis results attributed to the deoxygenation effect after torrefaction.

**Figure 7:**
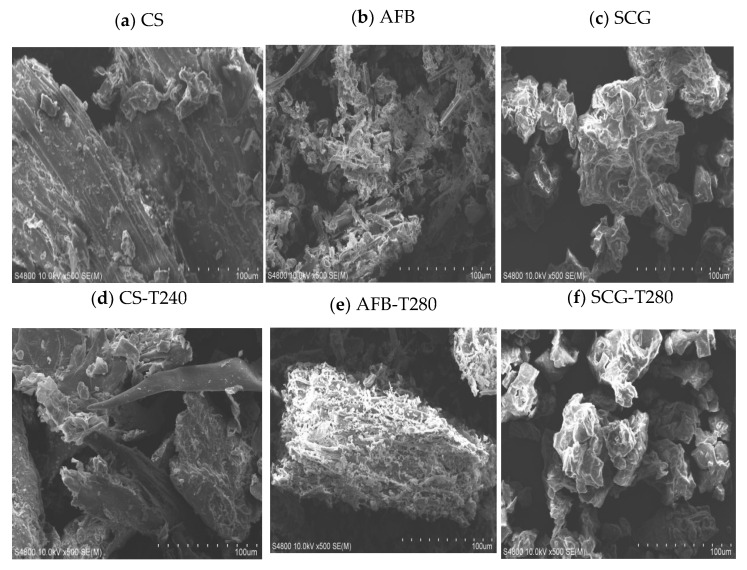
Microscopic morphology of raw biomasses and their optimal torrefied biochars.

**Figure 8:**
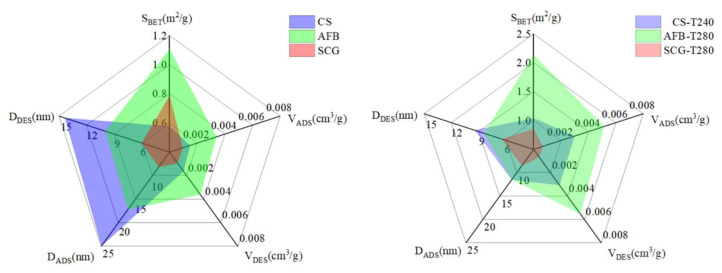
Specific surface area of raw biomasses and their optimal torrefied biochars.

Microstructure and specific surface area are important criteria for evaluating fuel quality from a microscopic perspective. The surface morphology and pore structure changes of CS, AFB, and SCGs, as well as their corresponding optimally torrefied biochar, were examined using SEM and a fully automatic specific surface area and BET. The results are detailed in Figure 7 and Figure 8. As shown in Figure 7a–c, there were significant differences in the microscopic morphologies of these three biomass raw materials. Compared to AFB and SCG, the surface structure of the CS raw material was smoother and more complete, whereas the AFB raw material exhibited trunk-like patterns with irregular blocky features lacking regularity and structure, and the SCG raw material presented irregular clumps. Under optimal torrefaction conditions, as shown in Figure 7d–f, significant changes occurred in the internal structures of CS, AFB, and SCGs. The surface of CS−T240 exhibited various small fragments and cracks resulting from the torrefaction-induced reduction in pore size, release of volatiles, and decomposition of organic matter [56], consistent with the BET observations. SCG−T280, due to the thermal decomposition of a large number of organic molecules in its structure, leads to the collapse of the pore walls, further reducing both the specific surface area and pore volume. In contrast, AFB−T280, owing to the heat treatment accompanied by the destruction of hemicellulose, partial decomposition of cellulose, and softening of lignin, causes tiny, dispersed particles to fuse, forming tubular structures, making it easier to grind. Simultaneously, there was a significant increase in both specific surface area and pore volume. In Figure 8, the specific surface area of these three raw biomass materials ranges from 0.5–1.2 m^2^/g and the pore volume ranges from 0.001–0.003 cm^3^/g. Under optimal torrefaction conditions, the specific surface area and pore volume increased to 1–2.21 m^2^/g and 0.002–0.005 cm^3^/g, respectively, which may be related to the raw materials. Through microscopic structural analysis, it is evident that torrefaction pretreatment has the most significant structural enhancement effect on AFB, and its optimal torrefied biochars have great potential as fuels or adsorbents [57].

### 2.4. Energy Consumption Calculation

**Figure 9:**
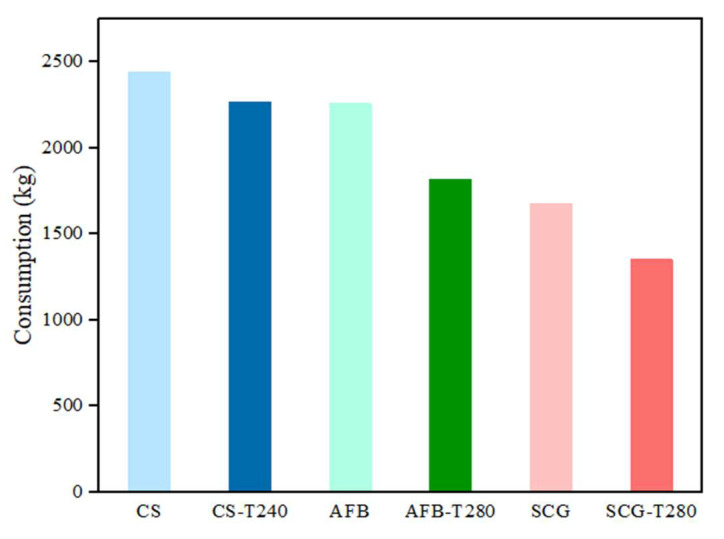
Energy consumption of raw biomass and their optimal torrefied biochars.

Taking a typical residential house in the northeastern region of China with an area of 120 m^2^ as an example, we calculated its energy consumption, as shown in Figure 9. Under the condition of an environmental temperature of −20 °C, the heat loss of the house was approximately 7200 W. Initially, the house relied on a coal-fired boiler for heating; however, this was replaced with a biomass fuel boiler, which provides heating for the home and supplies hot water, which is a significant factor affecting energy consumption. Based on the water usage habits of three typical families in the northeastern region, we calculated the hot water demand for the entire heating season. Combined with the heating days in Jilin Province (176 days), the total demand was 32,831 kWh. We conducted a survey and compared the economic costs. According to market price information from 2023, the price of ordinary mixed coal was as high as CNY 730 per ton, whereas the price of waste biomass raw materials was relatively affordable at only CNY 200 per ton. As the quantity of baked biomass fuel decreased by 7.03–19.32%, the cost decreased by 7.03–19.32%. Additionally, in the selection of biomass fuels, AFB-T280 showed the most significant reduction in consumption, whereas the consumption of the SCG-T280 samples was the lowest. These research findings provide households with a more economical and environmentally friendly choice for heating, helping reduce household energy expenditure, and promoting the popularization of renewable energy.

## 3. Materials and Methods

### 3.1. Materials Selection

This study selected the most representative biomass materials from Northeast China: CS, AFB, and SCGs, all sourced from Jilin City and the surrounding areas in the Jilin Province. To ensure particle uniformity, the raw materials were initially crushed and sieved to obtain uniformly sized particles in the range of 0–0.15 mm. Subsequently, the materials were dried in an oven at 105 °C for 24 h to remove any free moisture. Finally, the prepared CS, AFB, and SCG samples were stored in sealed airtight bags at room temperature for subsequent torrefaction experiments.

### 3.2. Torrefaction Experiments

**Figure 10:**
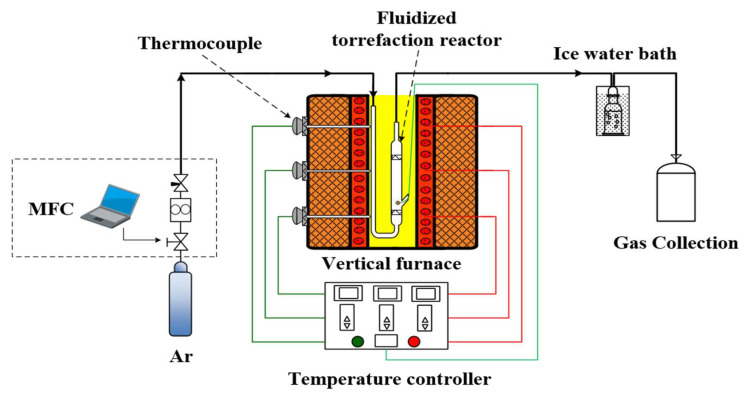
Schematic diagram of the experimental system.

The fluidized bed torrefaction experiments were conducted using a batch reactor system, as shown in Figure 10. In each experiment, 5 g of sample was introduced into the reactor, which was operated under an argon gas atmosphere at a pressure of 0.3 MPa, with a flow rate of 3 L/min selected from preliminary cold-state fluidization experiments. The reactor was heated from room temperature to the set torrefaction temperature (220, 240, 260, and 280 °C) at a constant heating rate of 10 °C/min. Once the pre-set temperature was reached, the samples were maintained at that temperature for 30 min. Throughout the experiment, the furnace temperature was measured using a thermocouple, and the central temperature of the reactor was recorded using a digital temperature sensor (Shanghai shengli Test Instrument, Shanghai, China) with an accuracy of 0.1 °C, with data recorded every 1 s. After the completion of the reaction, the carrier gas was immediately shut off, and the reactor was removed from the furnace and allowed to cool naturally to room temperature. Subsequently, the samples were removed and weighed up to 1 mg. For each char sample, three replicate experiments were conducted under identical conditions to ensure the accuracy of the experimental results. The samples were labeled according to the torrefaction temperature; for example, “CS-T220” indicates CS torrefied at 220 °C, with similar naming conventions for the other two biomass samples.

### 3.3. Analytical Method

#### 3.3.1. Basic Characteristics of Biomass Feedstock and Torrefied Biochar

Before experimentation, the samples were dried overnight at 105 °C. Subsequently, in accordance with Chinese standard GB/T30733-2014, the mass percentages of ash, volatile matter, and fixed carbon in the samples were measured using an industrial analyzer (SDLA718, Sandy, Changsha, China). According to the same standard, the mass percentages of carbon, hydrogen, and nitrogen in the samples were measured using an automatic elemental analyzer (EA3000, Euro Vector Company, Pavia, Italy). To measure the sulfur content, an infrared sulfur analyzer (SDS350, Sandy Company, Changsha, China) based on the Chinese standard GB/T25214-2010 was employed and the oxygen content was obtained using the difference method. According to the Chinese standard GB/T213-2008, the heating value was measured using an oxygen bomb calorimeter (SDC311, Sandy Company, Changsha, China). Additionally, the mass yield, energy yield, and energy density were calculated using Equations (1)–(3). Each set of samples was analyzed twice to enhance the accuracy of the experimental results, and average values were obtained [22].

(1) $$\mathrm{Mass}\;\mathrm{Yield}=\frac{\mathrm{Weight}\;\mathrm{of}\;\mathrm{torrified}\;\mathrm{biomass}}{\mathrm{Weight}\;\mathrm{of}\;\mathrm{raw}\;\mathrm{biomass}}\times 100\%$$

(2) $$\mathrm{Energy}\;\mathrm{Yield}=\mathrm{Mass}\;\mathrm{Yield}\times \frac{\mathrm{HHV}\;\mathrm{of}\;\mathrm{torrified}\;\mathrm{biomass}}{\mathrm{HHV}\;\mathrm{of}\;\mathrm{raw}\;\mathrm{biomass}}$$

(3) $$\mathrm{Energy}\;\mathrm{Density}=\frac{\mathrm{Energy}\;\mathrm{Yield}}{\mathrm{Mass}\;\mathrm{Yield}}$$

#### 3.3.2. Normalized Assessment of Various Biomass Torrefied Biochars

The devolatilization behavior of the biomass feedstock and torrefied chars was thoroughly investigated using a thermal gravimetric analyzer (Setline STA, Setaram Company, Caluire-et-Cuire, France). During the experiment, approximately 10 mg of each sample was placed in an alumina ceramic crucible. Subsequently, the sample was heated from ambient temperature to 850 °C at a rate of 10 °C/min under a nitrogen (N_2_) flow rate of 100 mL/min and maintained at this temperature for approximately 5 min. To optimize the evaluation of different types of torrefied biochar, we introduced an indicator called the “Displacement Level”, which comprehensively assesses fuel properties and allows for horizontal comparisons. This method calculates the overall sum of the absolute differences between the original thermogravimetric analysis (TGA) curve and the experimental TGA curve (DTG) within a specified temperature range (from the onset temperature of thermal decomposition, *T_s_*, to the final temperature, *T_f_*) (Equation (4)) [58].

(4) $$Displacement=\sum_{Ts}^{Tf}\left[\left|\left(\frac{dw}{dt}\right)b_{f,T}-\left(\frac{dw}{dt}\right)b_{c,T}\right|\right]$$

where *T*_s_ and *T*_f_ are the onset and final temperatures of decomposition (25–850 °C), respectively, and (*d_W_/d_t_*)*b_f,T_* and (*d_W_/d*_t_)*b_c,T_* are the weight loss rates of the biomass feedstock and torrefied biochar, respectively, at a specific temperature during pyrolysis.

#### 3.3.3. Microscopic Characterization of Biomass Feedstock and Optimal Torrefied Biochar

We conducted solid-phase infrared analysis using a Fourier-transform infrared spectrometer (FTIR; Perkin Elmer Company, Waltham, MA, USA) with a wavelength accuracy of 0.01 cm^−1^. A scanning electron microscope (SEM; JSM-7610F, JEOL, Akishima, Japan) was used to directly observe the microscopic structure of the samples. Furthermore, we used an automated adsorption apparatus (Brunauer–Emmett–Teller (BET); TriStarII3020, Micromeritics Company, Norcross, GA, USA) to measure the pore structure through nitrogen adsorption. Subsequently, we used the BET equation, Barrett–Joyner–Halenda model, and single-point adsorption total pore volume analysis to determine the BET-specific surface area (SBET), average pore diameter (D), and total pore volume (V).

#### 3.3.4. Energy Consumption of Biomass Feedstock and Optimal Torrefied Biochar

We calculated the total heat demand during the heating season using simulated household calculations for pellet consumption, based on Equation (5):

(5) $$Q_{h,year}=Q_{h,\max}\times \frac{t_{is}-t_{e^{\prime }}}{t_{is}-t_{e}}\times 3600\times 24\times i_{n}\times 10^{-6}$$

where *Q_h,year_* is the heat consumption during the heating season [MJ], *Q_h,_*_max_ is the maximum power required for the temperature in a given area [W], *t_i_*_s_ is the desired temperature in the interior [°C], *t_e_* is the average outdoor temperature in the heating season [°C], *t_e_*_′_ is the lowest temperature for the given area [°C], and *i_n_* is the number of days in the heating season [−].

The heat required to heat hot water each day for laundry, cleaning, hygiene, food and beverage preparation, and personal hygiene requires hot water. The amount of hot water consumed depends on the number of occupants, lifestyle, and the age of the household. Hot water will be prepared in a 160 L boiler, heating water from 10 °C to 55 °C. The daily heat required for hot water heating was calculated using Equation (6):

(6) $$Q_{hw}=1.15\times V_{w}\times cp_{w}\times (t_{h}-t_{c})\times 10^{-3}$$

where *Q_hw_* is the heat consumption for heating hot water [MJ], 1.15 is the system efficiency coefficient [−], *V_w_* is the quantity of heated water [l], *cp_w_* is the specific heat capacity of water [kg/(kg·k)], *t_h_* is the hot water temperature [°C], and *t_c_* is the cold water temperature [°C]. The heat input required for the boiler to heat the water was 5 h. The heat input required for the boiler was calculated using Equation (7):

(7) $$P_{t}=\frac{Q_{hw}}{t_{o}}$$

where *P_t_* is the required thermal input of the boiler to heat the hot water [kW] and *t_o_* is the heating time [h].

The heat required for hot water heating during the heating season was calculated using Equation (8):

(8) $$Q_{hw,year}=Q_{hw}\times i_{d}$$

The total heat demand was calculated as the sum of the heat required for heating and that required for hot water within a given heating period (Equation (9)):

(9) $$Q_{h,t}=Q_{h,year}+Q_{hw,year}$$

Pellet consumption was determined based on the calculated heat value ((Equation (10)):

(10) $$m_{fuel}=\frac{Q_{h,t}}{Q_{v}.\eta_{b}}$$

where is *m_fuel_* is the quantity of pellets [kg], *Q_v_* is the heat value of pellets [MJ/kg], and *η_b_* is the boiler efficiency [−].

## 4. Conclusions

This study comprehensively evaluated the fuel quality of CS, AFB, and SCG biomass using a novel integrated indicator called the “displacement level”. The results demonstrated that the torrefaction pretreatment process exhibited excellent performance for all three biomasses, not only increasing the fixed carbon (68.5–121%) and energy density (13.2–14.8%) of the biomass but also enhancing the combustibility and stability of the biomass fuel. The ranking of the optimal torrefied biochar fuel characteristics was as follows: AFB-T280 (260) > SCG-T280 (252) > CS-T240 (248). Torrefaction pretreatment had the most significant upgrading effect on AFB, increasing it by 152%, while the effect on SCGs was minimal, with only a 19% increase. Furthermore, microscopic structural analysis revealed that torrefaction pretreatment improved the microstructure and pore structure of the three biomass charcoal samples, particularly for AFB, with AFB-280 exhibiting increased hydrophobicity and grindability, with increases of 93.03% and 66.67% in specific surface area and pore volume, respectively. The results indicated that biomass fuel consumption depends on HHV. The biomass fuel consumption of the three optimally torrefied biochars decreased from 7.03 to 19.32%, with AFB-T280 showing the most significant reduction and SCG-T280 showing the lowest consumption. In conclusion, this study confirmed that AFB is the most suitable solid fuel for upgrading using torrefaction technology, demonstrating promising application potential. Future research should expand the sample range to include more types of biomass to comprehensively understand their torrefaction performance. Additionally, utilizing AFB produced under optimal torrefaction conditions to prepare pellets could provide practical guidance for achieving easier transportation, storage, processing, and conversion.

## Figures and Tables

**Table 1:** The mass yield, energy yield, HHV, and energy density of the three biomasses at different torrefaction temperatures.

Sample	Mass Yield (%)	Energy Yield (%)	HHV (MJ/kg)	Energy Density
CS	-	-	15.8	-
CS-T220	81.07	86.21	16.8	1.06
CS-T240	73.86	79.51	17	1.07
CS-T260	61.74	71.47	18.29	1.16
CS-T280	53.14	63.92	18.5	1.2
AFB	-	-	17.1	-
AFB-T220	87.76	96.15	18.74	1.09
AFB-T240	83.19	93.1	19.14	1.12
AFB-T260	72.99	83.95	19.67	1.15
AFB-T280	63.62	78.87	21.2	1.24
SCG	-	-	23.03	-
SCG-T220	85.88	92.92	24.92	1.08
SCG-T240	76.36	84.84	25.59	1.11
SCG-T260	64.63	76.36	27.22	1.18
SCG-T280	58.14	71.97	28.51	1.24

**Table 2:** Summary of existing research on biomass torrefaction optimization.

Biomass	Optimal Methods	Optimal Torrefaction Conditions	EY (%)	HHV (MJ/kg)	H/O, O/C	Author
Cotton stalks	Response surface methodology	305 °C, 32 min	64.00	19.70	1.00, 0.17	Kutlu et al. [42]
Olive waste	Numerical model	275 °C, 30 min	72.01	20.43	/	Jaime et al. [24]
Rick husk	/	360 °C, no retention time	56.31	19.71	0.83, 0.34	Majam et al. [43]
Oil palm frond	/	300 °C, 30 min	65.80	20.32	0.67, 0.11	Tasi et al. [44]
CS, AFB, and SCGs	Displacement level	240, 280, 280 °C and 30 min	73.86, 63.62, and 58.14	17.00, 21.1, and 28.51	1.23–1.43, 0.24–0.89	This study

## Data Availability

Data are contained within the article.
